# Identification and Functional Analysis of Two Mitoferrins, CsMIT1 and CsMIT2, Participating in Iron Homeostasis in Cucumber

**DOI:** 10.3390/ijms24055050

**Published:** 2023-03-06

**Authors:** Karolina Małas, Katarzyna Kabała

**Affiliations:** Department of Plant Molecular Physiology, Faculty of Biological Sciences, University of Wrocław, Kanonia 6/8, 50-328 Wrocław, Poland

**Keywords:** iron, mitochondria, mitochondrial carrier family, mitoferrin, cucumber

## Abstract

Mitochondria are one of the major iron sinks in plant cells. Mitochondrial iron accumulation involves the action of ferric reductase oxidases (FRO) and carriers located in the inner mitochondrial membrane. It has been suggested that among these transporters, mitoferrins (mitochondrial iron transporters, MITs) belonging to the mitochondrial carrier family (MCF) function as mitochondrial iron importers. In this study, two cucumber proteins, CsMIT1 and CsMIT2, with high homology to *Arabidopsis*, rice and yeast MITs were identified and characterized. *CsMIT1* and *CsMIT2* were expressed in all organs of the two-week-old seedlings. Under Fe-limited conditions as well as Fe excess, the mRNA levels of *CsMIT1* and *CsMIT2* were altered, suggesting their regulation by iron availability. Analyses using *Arabidopsis* protoplasts confirmed the mitochondrial localization of cucumber mitoferrins. Expression of CsMIT1 and CsMIT2 restored the growth of the *Δmrs3Δmrs4* mutant (defective in mitochondrial Fe transport), but not in mutants sensitive to other heavy metals. Moreover, the altered cytosolic and mitochondrial Fe concentrations, observed in the *Δmrs3Δmrs4* strain, were recovered almost to the levels of WT yeast by expressing CsMIT1 or CsMIT2. These results indicate that cucumber proteins are involved in the iron transport from the cytoplasm to the mitochondria.

## 1. Introduction

Iron is a micronutrient essential for plant cell metabolism. It is involved in important cellular processes, including photosynthesis and respiration, by acting as an electron carrier and cofactor for many enzymes. Therefore, iron deficiency is a limiting factor in plant growth and development [1]. To counteract iron limitations in the environment and to improve crop yields, Fe is provided to plants in fertilizers [2]. Under aerobic conditions, most of the iron in the soil is present in the Fe^3+^ (ferric) form, whose solubility, especially at high pH, is too low to ensure a sufficient supply for plants. Thus, to make iron more accessible, plants have evolved complex and precisely regulated mechanisms that enable its uptake, distribution and storage in tissues and cell compartments [3]. Plants have developed two main strategies for efficient Fe acquisition from soil solutions. *Arabidopsis* and other non-graminaceous plants reduce Fe^3+^ to more soluble Fe^2+^ (ferrous) ions under acidic conditions using both plasma membrane H^+^-ATPase and FRO2 (ferric reductase oxidase) activity (Strategy I) [4,5]. In graminaceous crops, such as corn, rice and wheat, chelation of Fe^3+^ ions by phytosiderophores, plant-derived small organic molecules, occurs (Strategy II) [2,3]. The reduced or chelated iron is then taken up into the cytoplasm via the plasma membrane transporters IRT1 (iron-regulated transporter, Strategy I) or YS/YSL (yellow stripe/yellow stripe-like) oligopeptide transporters (Strategy II), respectively [6,7,8,9]. The mechanisms governing iron uptake strategies from soils have already been thoroughly reviewed [2,3,10,11].

Inside the cell, iron is directed to organelles with high iron requirements, such as mitochondria and chloroplasts, as well as to vacuoles, which are generally considered to be the main storage compartments of excess Fe that can be mobilized when needed [12]. Both iron vacuolar sequestration and efflux into the cytoplasm are regulated by tonoplast transporters. Among them, the VIT1 (vacuolar iron transporter), VTLs (vacuolar iron transporter 1-like proteins) and FPN2/IREG2 (ferroportins) are involved in Fe accumulation, whereas NRAMP3 and NRAMP4 (natural resistance-associated macrophage proteins) mediate Fe export from these organelles [13,14,15,16]. In plants, chloroplasts can also accumulate the high levels of cellular iron necessary for chlorophyll synthesis, photosynthesis and Fe-S cluster assembly. Iron homeostasis in chloroplasts is controlled by the FRO7 oxidoreductase responsible for Fe^3+^ reduction, PIC1 (permease in chloroplasts) involved in Fe uptake and YSL proteins participating in Fe efflux [17,18,19]. Since Fe is required in respiration-related processes, mitochondria are considered as another significant iron sink within plant cells. Similar to chloroplasts, Fe levels are tightly regulated in these organelles. FRO3 and FRO8 oxidoreductases have been proposed to function in Fe^3+^ reduction in the mitochondrial membrane [20]. However, the knowledge of mitochondrial iron transporters remains limited. Available data suggest that members of the conserved mitochondrial carrier family (MCF) are responsible for iron flux from the cytoplasm to the mitochondria [1]. 

Mitochondrial carriers (MCs) are small proteins (approximately 30–35 kDa) that are mainly located in the inner mitochondrial membrane (IMM) [21,22]. Although MCs transport various substrates, including nucleotides, cofactors and metabolic intermediates to the mitochondrial matrix, they share conserved structural features, including six transmembrane domains. Their primary structure exhibits three repeated homologous regions containing two hydrophobic membrane-spanning segments [21,22]. MCF proteins are secondary transporters that usually act as antiporters and less often as uniporters or symporters [23]. The first MCF transporters responsible for iron import into the mitochondria, MRS3 and MRS4 (mitochondrial RNA splicing 3 and 4), were identified in yeast [24]. Homologous proteins were found in humans, *Drosophila*, zebrafish and rice, and were named mitoferrins (or mitochondrial iron transporters, MITs) [21]. Studies of rice MIT have confirmed that this protein functions as a mitochondrial Fe transporter. Moreover, the loss of MIT function was lethal, and reduced *MIT* expression resulted in impaired growth, suggesting that this protein is essential for proper rice development [25,26]. Recently, genes encoding mitoferrins have been identified and characterized in *Arabidopsis thaliana* (AtMIT1 and AtMIT2) [27] and potato (StMIT1) [28]. Both AtMIT1 and AtMIT2 are important for mitochondrial iron acquisition, act redundantly and are essential for embryogenesis [27]. On the other hand, StMIT1 plays a significant role in response to drought and salinity [28].

In the present study, we report the identification of mitoferrin-encoding genes in cucumber (*Cucumis sativus*); thus, for the second time, MIT transporters have been identified in dicot plants of significant agricultural importance. However, in contrast to potato and similar to *Arabidopsis*, two genes, *CsMIT1* and *CsMIT2*, are present in the cucumber genome. Here, we show that both cucumber CsMIT1 and CsMIT2 are mitochondrial iron transporters involved in Fe trafficking from the cytosol to the mitochondria. Our results also indicate that the expression of both genes is organ-specific and depends on Fe availability.

## 2. Results

### 2.1. Identification and Expression Analysis of CsMIT1 and CsMIT2

**Figure 1:**
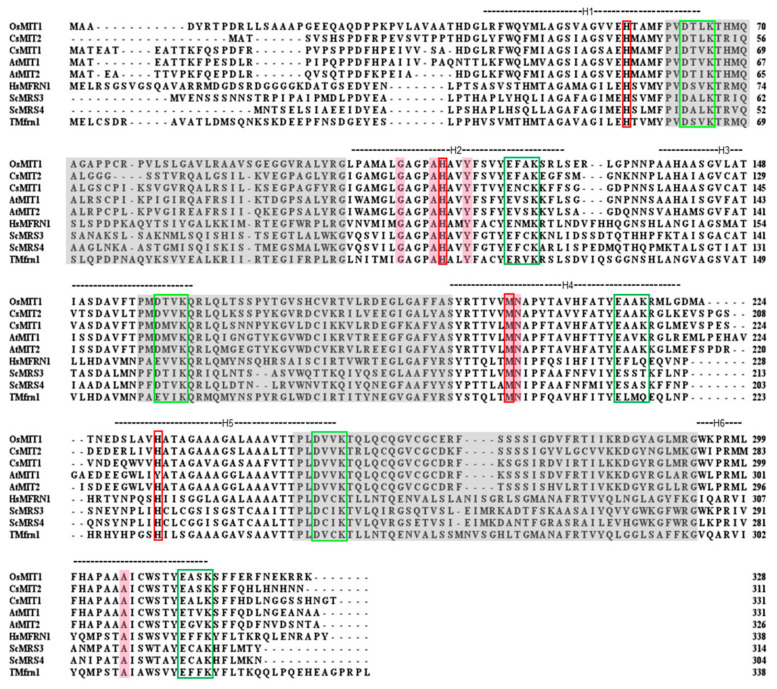
In silico analysis of MIT proteins from yeast, human, fish, and monocotyledonous and dicotyledonous plants. Sequence alignment of rice (OsMIT1), *Arabidopsis* (AtMIT1 and AtMIT2), cucumber (CsMIT1 and CsMIT2), human (HsMfrn1), yeast (ScMRS3, ScMRS4) and *O. niloticus* (TMfrn1) generated using Clustal O. Grey highlights represent the characteristic sequence motif of MCs (P-x-[DE]-x-[LIVAT]-[KR]-x-[LRK]-[LIVMFY]-(20-30 residues)-[DE]-G-x-x-x-x-[WYF]-[KR]-G). Transmembrane helices predicted for these proteins are depicted by H1-H6. Putative substrate contact sites are highlighted in pink, and the residues involved in iron transport are marked in red boxes. Green boxes represent the conserved motifs forming salt bridges on the cytosolic (dark green) and matrix (light green) side.

The full putative genomic sequences of *CsMIT* genes were identified and analyzed using *A. thaliana* cDNAs of MIT1 (At2g30160) and MIT2 (At1g07030) as the query sequences, cucumber (Borszczagowski cultivar) whole contigs (ACYN01002532.1 for CsMIT1 and ACYN01006329.1 for CsMIT2), and BLAST^®^ (blastn) program (NCBI server). Based on the FGENESH results, the genomic sequences of *CsMIT1* and *CsMIT2* contain two exons and one intron, encoding putative proteins of 331 and 311 amino acids in length and a calculated molecular weight of 35,74 and 33,17 kDa, respectively. Analysis of amino acid sequences revealed that both proteins have six transmembrane helices (TMHs), with their ends facing the intermembrane mitochondrial space (IMS), and no putative mitochondrial-targeting peptide (TargetP, Mitofates) [29,30]. Additionally, as members of the mitochondrial carrier family, these proteins exhibit, although with small divergence, well-established MCF features [31,32,33]: a highly conserved, characteristic sequence motif (P-x-[DE]-x-[LIVAT]-[KR]-x-[LRK]-[LIVMFY]-(20-30 residues)-[DE]-G-x-x-x-x-[WYF]-[KR]-G), contact points of the signature substrate-binding site on even-numbered helices and salt bridges on the IMS and matrix sides, defined by the [D/E]-X-X-[R/K] motif, hypothesized to facilitate substrate transport by MCs (Figure 1). Studies conducted on yeast MRS3 and MRS4 as well as on TMfrn1 from *Oreochromis niloticus* showed the importance of several amino acids in the transport activity, substrate binding, and protein folding of mitoferrins [34,35], which can also be found in CsMIT1 and CsMIT2 (Figure 1). 

**Figure 2:**
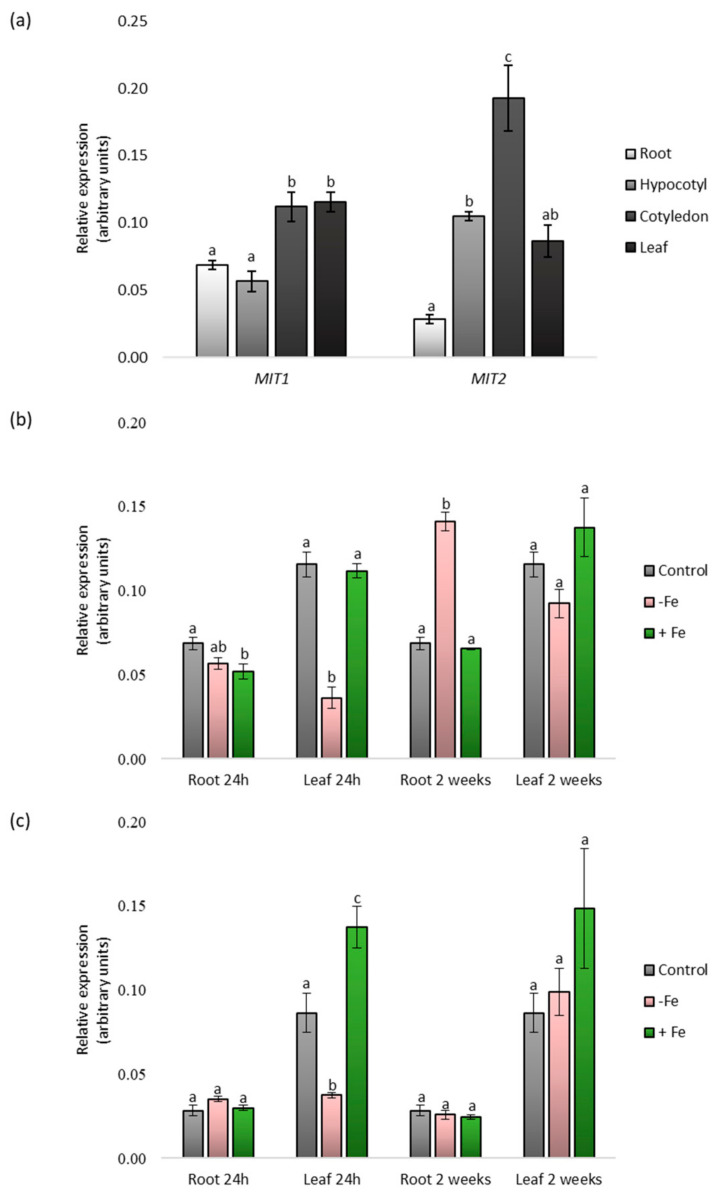
*CsMIT1* and *CsMIT2* gene expression in different organs of cucumber plants grown under various iron treatments. (**a**) The expression of *CsMIT1* and *CsMIT2* in the vegetative organs of two-week-old cucumber seedlings grown in control medium. (**b**) The expression of *CsMIT1* in the roots and leaves of two-week-old cucumber seedlings grown in control medium or subjected to Fe deficiency (-Fe) and Fe excess (+Fe) for 24 h or two weeks. (**c**) The expression of *CsMIT2* in the roots and leaves of two-week-old cucumber seedlings grown in control medium or subjected to Fe deficiency (-Fe) and Fe excess (+Fe) for 24 h or two weeks. For real-time analysis, expression of individual genes was calculated relative to the reference gene *CsCACS* according to the ΔΔCT method. Results shown are the means of three biological replicates; error bars represent standard error (+/-SE). Different letters represent statistically significant differences (*p* < 0.05; ANOVA with Tukey’s correction).

To determine the expression profiles of *CsMIT1* and *CsMIT2*, real-time PCR expression analysis was performed in roots, hypocotyls, cotyledons and first leaves, which were collected from two-week-old seedlings. As shown in Figure 2a, both transcripts were detectable in all vegetative organs but with different expression patterns. An approximately two-fold increase in *CsMIT1* transcript levels was observed in the cotyledons and leaves compared to that in the roots and hypocotyls. In contrast, increased *CsMIT2* expression was observed mainly in the cotyledons; it was approximately seven-fold and two-fold higher than that in the roots and hypocotyls/leaves, respectively. The lowest level of *CsMIT2* transcript found in the roots was more than three times lower than that in the hypocotyls and leaves. 

CsMIT1 and CsMIT2 mitoferrin homologs, characterized in yeast, rice and *Arabidopsis* [25,27,36], are proteins involved in the maintenance of iron homeostasis. Therefore, we verified whether the expression of genes encoding these proteins in *Cucumis sativus* was dependent on environmental iron availability. For this purpose, the plants were subjected to two different stress conditions: iron deficiency and iron excess in two time variants: short-term (24 h) and long-term (two weeks). Real-time PCR analysis was performed on the roots and first leaves. As indicated in Figure 2b,c, both *CsMIT1* and *CsMIT2* mRNA levels showed a decrease in leaves under 24 h iron limitation. In contrast, *CsMIT2* transcripts were moderately increased under iron excess in leaves treated with excess iron for 24 h. As a result of long-term treatment, the only significant change was observed for *CsMIT1* transcripts under iron deficit, with an increase in mRNA levels in roots, whereas *CsMIT2* mRNA levels were not affected by iron availability in the medium. Thus, our data indicate that the expression of cucumber *CsMIT1* and *CsMIT2* is regulated by Fe and is also organ-dependent.

### 2.2. Mitochondrial Localization of CsMIT1 and CsMIT2 in Arabidopsis thaliana Protoplasts

**Figure 3:**
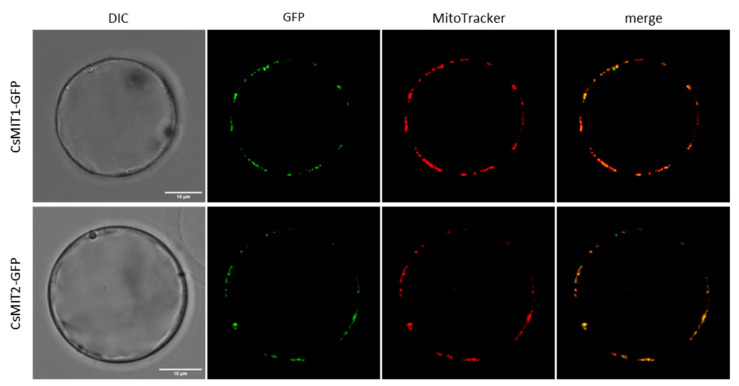
Subcellular localization of CsMIT1 and CsMIT2 in *A. thaliana* protoplasts. DIC—transmission images of the protoplasts expressing CsMIT1-GFP or CsMIT2-GFP, GFP—GFP fluorescence of the protoplast, MitoTracker—fluorescence of mitochondria specific marker. The scale corresponds to 10 μm.

Sequence analysis of CsMIT1 and CsMIT2 using DeepMito [37] showed, with high confidence, that both proteins localize to the IMM. To confirm the data obtained by in silico analysis, the subcellular localization of CsMIT1 and CsMIT2 was assayed *in vivo* using *A. thaliana* protoplasts. For this purpose, protoplasts were transiently transformed with a vector carrying the sequences encoding CsMIT1 or CsMIT2 proteins, driven by the 35S promoter and fluorescently tagged with GFP (CsMIT1-GFP and CsMIT2-GFP). Transformed protoplasts were stained with the vital dye MitoTracker Red and analyzed using a confocal microscope. As shown in Figure 3, the fluorescence of the CsMIT1-GFP and CsMIT2-GFP fusion proteins clearly colocalized with regions stained with MitoTracker, thus confirming their mitochondrial localization.

### 2.3. Effect of CsMIT1 and CsMIT2 Expression on the Metal-Sensitive Yeast Mutants

**Figure 4:**
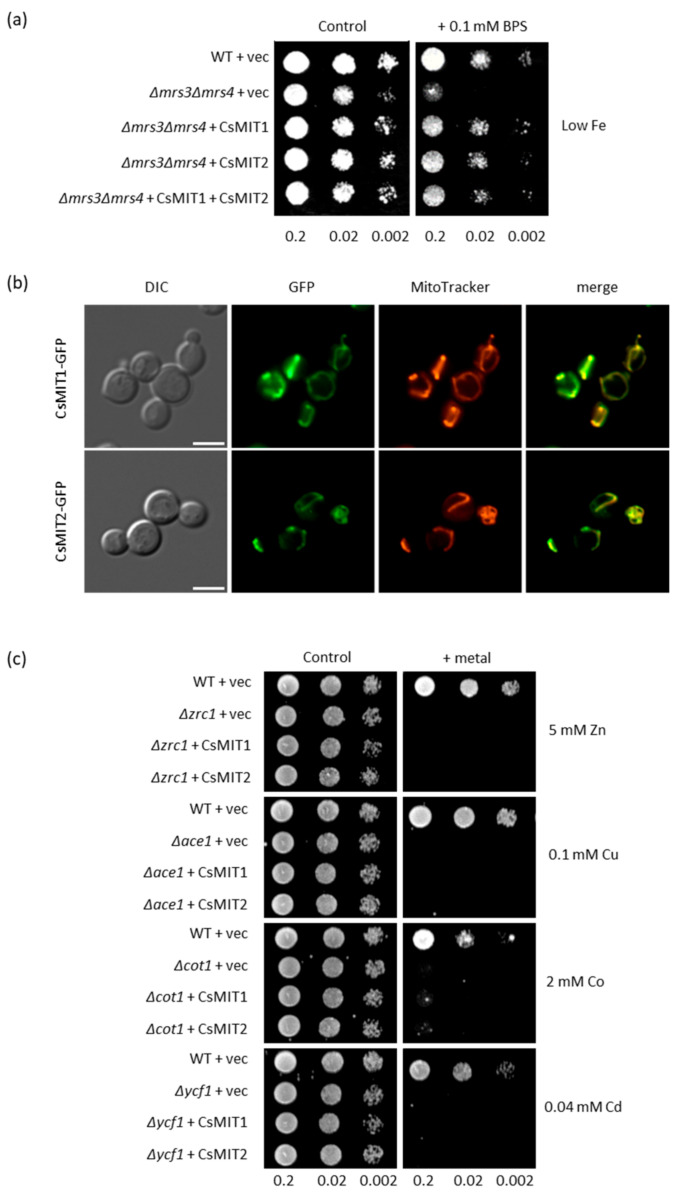
Effect of CsMIT1 and CsMIT2 expression in yeast. (**a**) Representative of serial dilutions corresponding to OD of 0.2; 0.02 and 0.002 of WT and *Δmrs3Δmrs4* cells transformed with empty vector or vector carrying CsMIT1 or/and CsMIT2 placed onto control SC/Glu-His medium or SC/Glu-His low-Fe medium containing 0.1 mM BPS. (**b**) Localization of CsMIT1 and CsMIT2 in *Δmrs3Δmrs4* cells. DIC—transmission images of the cells expressing CsMIT1-GFP or CsMIT2-GFP, GFP—GFP fluorescence of the cell, MitoTracker—fluorescence of mitochondria specific marker. The scale corresponds to 5 μm. (**c**) Representative of serial dilutions corresponding to OD of 0.2; 0.02 and 0.002 of WT and yeast mutants sensitive to various heavy metals. Cells were transformed with empty vector or vector carrying CsMIT1 or CsMIT2 placed onto control SC/Glu-His medium or SC/Glu-His medium supplemented with suitable metal.

To determine the potential metal substrates for CsMIT1 and CsMIT2, a heterologous system with *Saccharomyces cerevisiae Δmrs3Δmrs4* strain was used. The yeast homologues MRS3 and MRS4 function as mitochondrial iron importers. Deletion of the genes encoding both proteins renders this strain highly sensitive to low Fe conditions due to impaired iron transport to the mitochondria [38]. To verify whether cucumber proteins are involved in iron transport similar to their yeast orthologs, genes encoding CsMIT1 and CsMIT2 were cloned separately into yeast expression vectors, forming CsMIT1-GFP and CsMIT2-GFP fusion proteins. The double mutant strain was then transformed with the prepared constructs, and the empty vector was used as a negative control, with the wild-type strain serving as a positive control. As shown in Figure 4a, CsMIT1 and CsMIT2 expression in yeast restored the growth of the *Δmrs3Δmrs4* mutant under Fe-limited conditions. Moreover, the simultaneous expression of both proteins did not significantly enhance *Δmrs3Δmrs4* mutant growth relative to the expression of only one of them, suggesting that MIT proteins can function redundantly. 

The fluorescence of the CsMIT1/CsMIT2-GFP-expressing *Δmrs3Δmrs4* cells stained with MitoTracker Red showed colocalization of MitoTracker and GFP signals, confirming that CsMIT1 and CsMIT2 proteins are targeted to the mitochondria of yeast cells (Figure 4b). This, together with the complementation of MRS3 and MRS4 function by CsMIT1 and CsMIT2, indicates that both cucumber proteins are capable of delivering Fe to the mitochondria of yeast cells under Fe-limiting conditions.

Both rice and *Arabidopsis* MIT proteins have been reported to transport iron, whereas their animal homologues exhibit broader substrate specificity [35]. To verify whether other metals can be used as substrates by CsMIT1 and CsMIT2, cucumber proteins were expressed in yeast mutants that are sensitive to various heavy metals (Cu, Cd, Co and Zn). As shown in Figure 4c, the expression of CsMIT1-GFP or CsMIT2-GFP did not restore the growth of any of the mutants, suggesting that neither mitoferrin participates in the detoxification of yeast cells from other heavy metals.

### 2.4. Effect of CsMIT1 and CsMIT2 Expression on the Cytosolic and Mitochondrial Fe Content in Δmrs3Δmrs4 Cells

**Figure 5:**
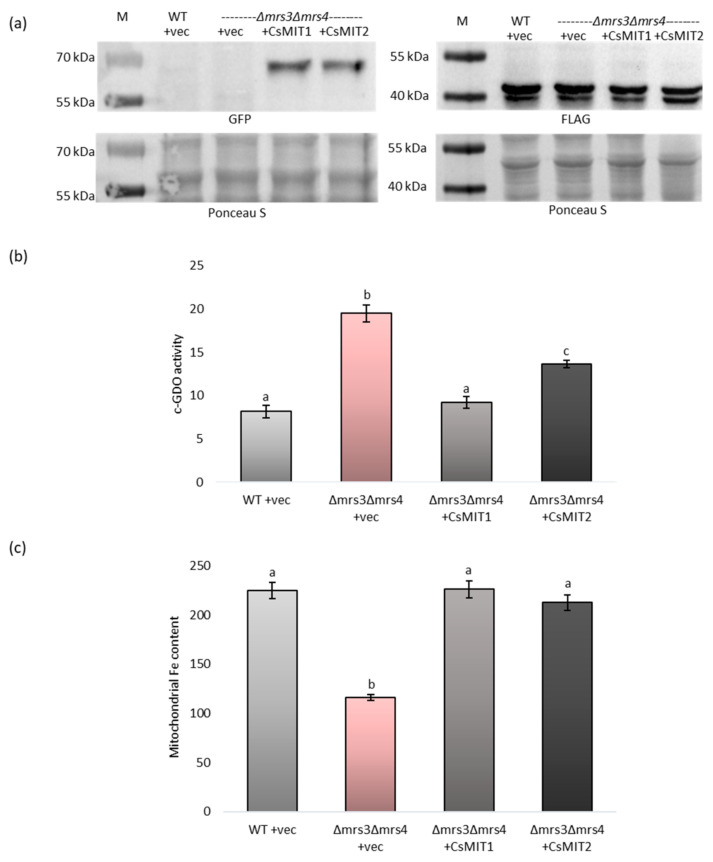
Cytosolic and mitochondrial Fe content in yeast expressing CsMIT1 and CsMIT2. (**a**) Western blot analysis of CsMIT1-GFP and CsMIT2-GFP (left) or c-GDO-FLAG (right) proteins in yeast *Δmrs3Δmrs4* transformants using the antibodies against GFP and FLAG, respectively. (**b**) c-GDO activity in WT and *Δmrs3Δmrs4* cells expressing c-GDO-FLAG and either empty vector, CsMIT1-GFP or CsMIT2-GFP. c-GDO activity is expressed as nanomoles of substrate converted per minute per mg of protein. Results shown are the means of four biological replicates; error bars represent standard error (+/-SE). Different letters represent statistically significant differences (*p* < 0.05; ANOVA with Tukey’s correction). (**c**) Mitochondrial Fe content in WT and *Δmrs3Δmrs4* cells expressing either empty vector, CsMIT1-GFP or CsMIT2-GFP. Results as shown are expressed as the mean Fe level of three biological replicates (nmol mg-1 protein); error bars represent standard error (+/-SE). Different letters represent statistically significant differences (*p* < 0.05; ANOVA with Tukey’s correction).

The obtained results suggest that CsMIT1 and CsMIT2 may be involved in iron transport between the cytoplasm and the mitochondria. To confirm this hypothesis, cytosolic and mitochondrial iron contents were measured in the *Δmrs3Δmrs4* strain with impaired mitochondrial iron loading. To assess cytosolic iron levels, the activity of gentisate 1,2-dioxygenase (c-GDO), a bacterial Fe-dependent enzyme, expressed in yeast under the control of the ADH1 promoter, was measured. c-GDO assays have been successfully used to determine Fe levels in this strain [39]. A plasmid carrying c-GDO-FLAG was introduced into both the wild-type and *Δmrs3Δmrs4* strains previously transformed with an empty vector and into a *Δmrs3Δmrs4* strain expressing CsMIT1-GFP or CsMIT2-GFP. The expression of all the proteins was confirmed by Western blot analysis (Figure 5a).

As indicated in Figure S1 and Figure 5a, immunoblotting with antibodies raised against the GFP protein showed that the apparent molecular masses of CsMIT1 and CsMIT2 were between 55 and 70 kDa, which is in accordance with those calculated for both GFP-tagged proteins (~63 kDa for CsMIT1 and 60 kDa for CsMIT2). Analysis of c-GDO activity (Figure 5b) showed that the iron level was two times higher in the cytoplasm of the *Δmrs3Δmrs4* cells transformed with the empty vector than in the wild-type cells, whereas the *Δmrs3Δmrs4* strain expressing the CsMIT1-GFP and CsMIT2-GFP fusion proteins exhibited cytosolic iron levels comparable to those of the wild-type strain. In contrast, iron levels were two times lower in mitochondria of *Δmrs3Δmrs4* cells transformed with the empty vector than in wild-type cells (Figure 5c). As with the c-GDO assay, the *Δmrs3Δmrs4* strain expressing CsMIT1-GFP and CsMIT2-GFP fusion proteins exhibited mitochondrial iron levels comparable to those of the wild-type strain (Figure 5c). Taken together, these results strongly indicate that both proteins participate in the transport of iron from the cytoplasm to the mitochondria.

## 3. Discussion

Mitochondria require iron for the biosynthesis of two fundamental factors that are ubiquitously present in cells: Fe-S clusters and heme. However, the processes involved in delivering Fe to plant mitochondria have not been fully elucidated. In *Arabidopsis*, two putative mitochondrial metalloreductases, FRO3 and FRO8, may play a role in mitochondrial Fe accumulation. Although there are indications of their role in iron trafficking, in-depth functional characterization of these proteins is still lacking [20,40,41].

Mitochondria consist of a matrix surrounded by both an IMM and OMM (outer mitochondrial membrane) separated by an IMS. Since the OMM is not selective toward small ions and molecules, their delivery into the matrix is regulated by transporters of the IMM. Among all the protein families present in the IMM, MCF, found in all eukaryotes, transports most of the substrates used in the metabolism of mitochondria [21,42]. As such, mitoferrins, which belong to the MCF, are good candidates for the role of Fe transporters. Studies using yeast and animal cells have indicated that they are located in the IMM [43]. 

Mitoferrins were first discovered in *Saccharomyces cerevisiae* and described as MRS3 and MRS4 proteins [24]. In yeast, deletion of *MRS3* and *MRS4* suppresses mitochondrial iron accumulation. Moreover, under cytosolic iron deficiency, this mutation impairs the Fe metabolism and biogenesis of mitochondrial Fe-S clusters, confirming that both carriers play an important role in mitochondrial iron acquisition [38,44]. 

In mammalian cells, two proteins, mitoferrin 1 (MFRN1) and mitoferrin 2 (MFRN2), were identified. They are functionally complementary to yeast MRS3 and MRS4 and are involved in iron transport across the IMM [45]. Notably, although both proteins seem to function redundantly, when there is a high demand for mitochondrial iron, mitoferrin-1 is crucial [46]. Moreover, it was found that MFRN1 physically interacts with the ABCB10 (ATP-binding cassette subfamily B) transporter, and this interaction stabilizes its function and enhances the mitochondrial import of Fe [47,48]. 

In plant organisms, the biochemical properties of MITs have been less studied, and for this reason, these proteins are less known [21]. Therefore, understanding their functions in plants is an important and current research topic. The first plant MIT-encoding gene was cloned and characterized in monocot rice. It has been demonstrated that the identified protein localized to the mitochondria when expressed as MIT-GFP in tobacco BY-2 cells. In addition, *MIT* complemented the growth defects of the yeast *Δmrs3Δmrs4* mutant. On the other hand, the rice *mit* knockout mutant *mit-1* was characterized by a lethal phenotype, while knockdown mutant *mit-2* exhibited a slow growth phenotype with reduced chlorophyll content [25,26]. Since this discovery, homologs of yeast and rice mitoferrins have been found in the genomes of two dicot plants, AtMIT1 and AtMIT2 in *Arabidopsis*, and StMIT in potato [27,28]. In this study, two orthologs of yeast and *Arabidopsis* mitoferrins, *CsMIT1* and *CsMIT2*, were identified in the genome of cucumber (*Cucumis sativus*), which is the third most-produced vegetable in the world according to Statista (statista.com, data from 2021). Considering the agricultural aspect, research on cucumber is crucial to understand the mechanisms responsible for the acquisition and homeostasis of minerals, such as iron, by crops.

All MCF proteins identified thus far have three homologous repeats (~100 amino acids each) and exhibit a highly conserved, characteristic sequence motif in each of these repeats. In addition, alignment of the sequences of proteins from individual groups, divided according to the transported substrate, allowed for identification of the so-called contact points. These amino acids separated between three substrate-binding sites, present on even-numbered helices, are specific for each of the designated groups [31]. All characteristic features were also found in CsMIT1 and CsMIT2, confirming that both proteins belong to the MCF family. These features include amino acids G107, A108, H112, and Y115 within H2, M198 and N199 within H4, A306 within H6 for CsMIT1 as well as G91, A92, H96, and Y99 within H2, M182 and N183 within H4, A290 within H6 for CsMIT2 at contact points I, II and III, respectively. Moreover, salt bridges on the IMS and matrix sides, commonly referred to as matrix and cytosolic gates [32], are also conserved in cucumber proteins. Phylogenetic analyses suggested differences between MIT proteins from monocot (rice, maize) and dicot *(Arabidopsis*, potato) plants and clade-specific motifs [28]. However, the dicot-specific motif proposed by Kurt et al. [28] is present entirely in CsMIT1 but only partially in CsMIT2. To establish whether the motifs specific to monocotyledonous and dicotyledonous plants are present in mitoferrins, as well as to determine their precise sequences, it is necessary to perform an extensive analysis of those proteins from multiple plant species.

*Arabidopsis* mitoferrins, which localize to mitochondria, share 81% amino acid sequence identity with each other and only 38% with yeast proteins. Their expression in yeast restores the *Δmrs3Δmrs4* mutant phenotype. *Arabidopsis mit1* and *mit2* single mutants show no apparent phenotypic changes, whereas the double mutation results in embryo lethality. Using a *mit1−−/mit2+−* double mutant, it was shown that the loss of both mitoferrins is associated with a decrease in mitochondrial iron and, consequently, mitochondrial dysfunction [27] suggesting the key role of these proteins in Fe trafficking to plant mitochondria. Using heterologous expression of CsMIT1-GFP/CsMIT2-GFP in *A. thaliana* protoplasts and yeast cells, as well as an immunodetection in the latter, we revealed that both cucumber proteins (which show approximately 65–76% amino acid sequence similarity to *Arabidopsis* mitoferrins) are also targeted to mitochondria. In addition, CsMIT1 and CsMIT2 improve the ability of the *Δmrs3Δmrs4* yeast mutant to grow under Fe-deficient conditions, and the expression of a single cucumber protein, same as *Arabidopsis* or mammalian mitoferrins, is sufficient to restore the mutant phenotype. The obtained results confirmed that the CsMIT1 and CsMIT2 proteins are involved in mitochondrial iron homeostasis. In addition, they can act redundantly like AtMIT1 and AtMIT2. This feature, distinguishing cucumber from potato, in which one StMIT protein is present, seems to be extremely important for plant adaptation to environmental conditions differing in mineral content.

As demonstrated in *Arabidopsis* and rice, plant mitoferrins are ubiquitously expressed. *MIT* transcripts were found in all examined organs and stages of plant development [25,27]. Similarly, in cucumber, both *CsMIT1* and *CsMIT2* transcripts have been detected in all vegetative organs, including roots, hypocotyls, cotyledons and leaves; however, their levels differ depending on the organ. Similarly, mammalian mitoferrins also exhibit tissue-specific expression, with mitoferrin-1 being predominantly expressed in erythroid tissues [45].

Analysis of the expression profile of rice *MIT* indicated that the transcription of this gene is strongly regulated by iron availability. Under Fe-limited conditions, transcript levels were markedly lower in both the roots and shoots than in control plants [25]. In contrast, in *Arabidopsis*, the expression of both *AtMIT1* and *AtMIT2* was decreased in the shoots, but not in the roots, of two-week-old seedlings grown under Fe-deficient conditions compared to plants that were provided with sufficient Fe [27]. A similar expression pattern was observed for cucumber genes under short-term Fe-deficiency conditions. The transcript levels of both *CsMIT1* and *CsMIT2* were significantly lower in the leaves but not in the roots of cucumber. Under long-term Fe shortage, only the level of *CsMIT1* noticeably increased in roots, suggesting its possible role in iron acquisition under deficiency conditions. 

On the other hand, the rice *MIT* levels increased in the roots and shoots of plants exposed to excess Fe [25]. In cucumber, no significant changes in *CsMIT1* expression were found in the roots and leaves of plants treated with high levels of iron. In contrast, under short-term Fe excess, *CsMIT2* transcript levels increased in the leaves. These results suggest that *CsMIT1* and *CsMIT2* expression is organ-specific and is regulated differently by iron. In turn, each of the two proteins may be specialized toward adaptation to different iron availability conditions. 

Although mitoferrins have been demonstrated to be high-affinity or high-throughput iron transporters, studies in yeast and animals have suggested that they can transport ions other than iron across the membrane. Mühlenhoff et al. [38] proposed that *S. cerevisiae* mitoferrins may also participate in the mitochondrial uptake of zinc, and possibly manganese and cobalt. TMfrn1 from the fish *O. niloticus* binds with micromolar affinity not only Fe^2+^, but also Mn^2+^, Co^2+^, and Ni^2+^. Moreover, the activity of TMfrn1 reconstituted in proteoliposomes showed that in addition to iron, it can transfer manganese, cobalt, copper and zinc across the membrane, but not nickel [35]. As reported previously, deletion of MRS3 and MRS4 in *Δmrs3Δmrs4* resulted in decreased cytosolic Fe, with a simultaneous increase in vacuolar Fe and the induction of a low-Fe transcriptional response [39]. Our results showed that mitochondria isolated from the *Δmrs3Δmrs4* yeast mutant expressing cucumber genes accumulated iron at a level similar to that of the wild-type strain. Similarly, using c-GDO activity, it was demonstrated that the high cytosolic Fe concentration, observed in the mutant cells, decreased to nearly WT levels in yeast transformed with *CsMIT1* or *CsMIT2*, confirming that cucumber mitoferrins are involved in iron transport from the cytoplasm to the mitochondria. On the other hand, the growth of heavy metal-sensitive yeast mutant strains expressing cucumber genes was not restored when Cu, Zn, Cd or Co was supplied, suggesting that CsMIT1 or CsMIT2 function exclusively as iron transporters. However, such a point of view requires further study. 

The mechanism of iron transport via mitoferrins remains an open question. Three His residues, which are highly conserved and essential for yeast MRS3 and MRS4 function, have been identified. A hypothetical model was proposed in which these three residues form a ladder of Fe-binding ligands across the carrier cavity. The authors suggested that His residues might have a specific role in iron transport, as they are not conserved in other members of the MCF family [34]. Likewise, mutagenesis-based analysis of TMfrn1 from *O. niloticus* indicated that mutations in histidine, cysteine and methionine residues had the greatest impact on the transport function of TMfrn1, confirming that aa residues with side chains that can coordinate iron ions, especially imidazole groups, may act as metal-binding sites or may interact during substrate translocation [35]. The three histidines identified by Brazzolotto et al. [34] in yeast mitoferrins, as well as M202 from TMfrn1 [35], are conserved in CsMIT1 and CsMIT2, pointing to their potential to transport iron across the mitochondrial membrane. It has also been reported that this transport is affected by the transmembrane pH gradient, but iron translocation itself is not coupled with proton movement [35]. Furthermore, these studies suggest that TMfrn1 carries free Fe ions and not a chelated Fe complex across the membrane [35]. In contrast, citric acid was proposed by Kurt et al. [28] as a ligand for Fe transport catalyzed by MITs. In StMIT, amino acid residues that interact with the citrate–Fe complex have been proposed [28]. It seems very interesting and important to explain which of the proposed forms of iron is an actual substrate for mitoferrins.

The results of previous studies indicated that mitoferrins are involved in plant growth and development as well as in plant reactions to stress factors. Silencing of AtMIT1 and AtMIT2 expression results in enhanced uptake of Zn^2+^ and other divalent microelements in *Arabidopsis*. Both mitoferrins are essential for plant metabolism under Fe-deficient conditions as well as during embryogenesis [27]. Mitochondrial Fe deficiency related to impaired mitoferrin function not only differentially affects gene expression patterns in rice roots and shoots, but also reprograms the whole metabolism in plant tissues [49]. In potato, increased *StMIT* expression was observed in the roots and leaves of plants grown under drought and salinity conditions, suggesting an important role of mitoferrins in plant responses to abiotic stress [28]. As shown in this study, the expression of cucumber mitoferrins is regulated by environmental iron availability. However, it remains to be resolved in the future whether common abiotic stress conditions other than Fe availability may directly or indirectly affect the gene or protein expression of CsMIT1 and CsMIT2, indicating their crucial function in plant adaptation mechanisms.

## 4. Materials and Methods

### 4.1. Plant Material and Growth Conditions

Cucumber plants (*Cucumis sativus* var. Krak) were cultivated for two weeks on standard hydroponic medium [50] and were either supplemented with 1 mM FeSO_4_-EDTA or deprived of Fe by adding 30 µM bathophenanthroline disulfonate (BPS, Merck KGaA, Darmstadt, Germany), a Fe chelator, to the medium solution lacking Fe. Seedlings were grown under a 16 h/8 h day/night regime (180 µmol m^−2^ s^−1^) in a controlled environment at 24 °C.

### 4.2. RNA Extraction and Real-Time PCR

Total RNA was extracted from cucumber vegetative organs using Extrazol (Blirt, Gdańsk, Poland), according to the manufacturer’s instructions. The samples were then reverse-transcribed using a high-capacity cDNA synthesis kit (Applied Biosystems, Foster City, CA, USA). Real-time PCR was performed with primers specific for *CsMIT1* and *CsMIT2* in a Lightcycler 480 (Roche, Basel, Switzerland) using RealTime 2x PCR SYBR Mix (A&A Biotechnology, Gdańsk, Poland) under the following conditions: 95 °C for 30 s, followed by 45 cycles of 95°C for 10 s, 60 °C for 10 s and 72 °C for 15 s. Amplifications were normalized to the reference gene encoding the clathrin adaptor complex subunit (*CsCACS*) [51], and relative gene expression values were calculated using the ΔΔC_T_ method. The primer sequences used are listed in Table S1.

### 4.3. Cloning of CsMIT1 and CsMIT2

cDNA synthetized from DNase-treated RNA corresponding to *CsMIT1* and *CsMIT2* was cloned by PCR amplification. For heterologous expression in the yeast *Saccharomyces cerevisiae*, coding sequences of *CsMIT1* and *CsMIT2* genes were amplified from the cDNA as the XbaI-EcoRI fragment for CsMIT1 and EcoRI-SalI fragment for CsMIT2. The amplified fragments were then subcloned into the XbaI-EcoRI or EcoRI-SalI sites of the yeast expression vectors pUG23 or pUG35 [52]. For the protein localization assay in *Arabidopsis thaliana* protoplasts, *CsMIT1* and *CsMIT2* cDNA were subcloned into SpeI-SpeI or SalI-SpeI sites of the pA7-GFP vector [53]. The sequences of the primers used are listed in Table S1, and the vectors used are listed in Table S2.

### 4.4. Yeast Strains, Media and Fluorescence Imaging

The wild-type and deletion strains used in this study are listed in Table S2 [54,55,56]. YPD medium (containing 2% (*w*/*v*) glucose (POCH, Gliwice, Poland), 2% (*w*/*v*) bactopeptone (BD, Sparks, MD, USA) and 1% yeast extract (BD, Sparks, MD, USA) was used to maintain the strains. Complete synthetic media lacking histidine (SC-His), uracil (SC-Ura) or both (SC-His-Ura), containing 0.67% (*w*/*v*) yeast nitrogen base without amino acids (BD Difco, Sparks, MD, USA), 2% (*w*/*v*) glucose, the required amino acids and 2% (*w*/*v*) agar (BD Difco, Sparks, MD USA) were used for the selection of transformants. Fluorescence images of the protein localization of CsMIT1 and CsMIT2 in yeast mitochondria were acquired using an Axio Imager M2 Fluorescence Microscope (Carl Zeiss, Oberkochen, Germany) equipped with a 100x oil-immersion objective. The mitochondria were stained with 100 nM MitoTracker Red CMXRos (Invitrogen, Molecular Probes, Eugene, OR, USA) for 60 min prior to detection.

### 4.5. Metal Sensitivity Spot Assays

Yeast cells were grown overnight, and 5 µL of serial dilutions (OD_600_ 0.2; 0.02; 0.002) were spotted onto agar plates containing selective media supplemented with 100 µM BPS or different concentrations of heavy metals as indicated in the figures. The plates were incubated for 3–5 days at 30 °C.

### 4.6. Cytosolic Gentisate 1,2-Dioxygenase Assays

Vector containing Gentisate 1,2-dioxygenase (GDO) was a generous gift from Professor J. Kaplan (University of Utah, Salt Lake City, UT, USA). The plasmid was transformed into yeast cells harboring the pUG23, pUG23-CsMIT1 or pUG23-CsMIT2 plasmids, and transformants carrying both vectors were selected on SC/Glu-Ura-His solid media. Cells were grown in SC/Glu-Ura-His liquid medium overnight to mid-log phase and then for 12 h in fresh medium supplemented with 100 µM BPS. Cell lysates were prepared using glass bead homogenization, as described previously [57]. The presence of c-GDO in the yeast extracts was confirmed by Western blot analysis using antibodies against FLAG (1:10,000; Sigma Aldrich, St. Louis, MO, USA). The activity of the c-GDO enzyme was measured spectrophotometrically at 340 nm, as described previously [57], and was calculated using an extinction coefficient of 10.2 cm^−1^ mm^−1^. c-GDO activity was expressed as nmol of substrate converted per minute per mg of protein.

### 4.7. Preparation of Mitochondrial Fraction from Yeast Cells

To determine the metal content in yeast mitochondria, cells transformed with either the empty pUG23 vector or pUG23 plasmid carrying *CsMIT1* or *CsMIT2* were grown for 48 h in SC/Glu-His medium and then for 12 h in the same medium supplemented with 100 µM BPS. Mitochondrial fractions were prepared as previously described [58]. The presence of CsMIT1 and CsMIT2 in the mitochondrial fraction was confirmed by Western blot analysis using antibodies against GFP (1:5000, Roche, Basel, Switzerland).

### 4.8. Determination of Mitochondrial Heavy Metal Content

Mitochondria extracted from yeast cells were digested with 5 mL of concentrated HNO_3_ at 160 °C for 2 h. The iron content was measured using flame atomic absorption spectrometry (AAS, Perkin-Elmer Inc., Waltham, MA, USA).

### 4.9. Protein Determination

The protein contents of the cytosolic and mitochondrial fractions were determined using the Bradford assay [59].

### 4.10. Transformation of Arabidopsis thaliana Protoplasts and Confocal Imaging

Isolation and transformation of *Arabidopsis thaliana* protoplasts from cell suspensions were performed as previously described [60]. The protoplasts were then incubated for two days in the dark at 23 °C. Confocal fluorescence images were acquired using an inverted Leica TCS-SP8 confocal laser scanning microscope (Leica Microsystems, Wetzlar, Germany) at 24–36 h after transformation. Mitochondria were stained with 100 nM MitoTracker Red CMXRos for 60 min prior to detection.

### 4.11. Bioinformatics and Accession Numbers

Putative transmembrane domains were predicted using CCTOP [61]. Sequence alignment of plant, yeast, fish and human mitoferrins was performed using Jalview and Clustal O [62]. The nucleotide sequences of *CsMIT1* and *CsMIT2* have been submitted to GenBank under accession numbers OP454140 (CsMIT1) and OP454141 (CsMIT2).

### 4.12. Statistical Analysis

Tukey’s test and analysis of variance (ANOVA) were used for statistical analysis (Statistica 13.3, TIBCO Software Inc., Palo Alto, CA, USA; 2017).

## 5. Conclusions

**Figure 6:**
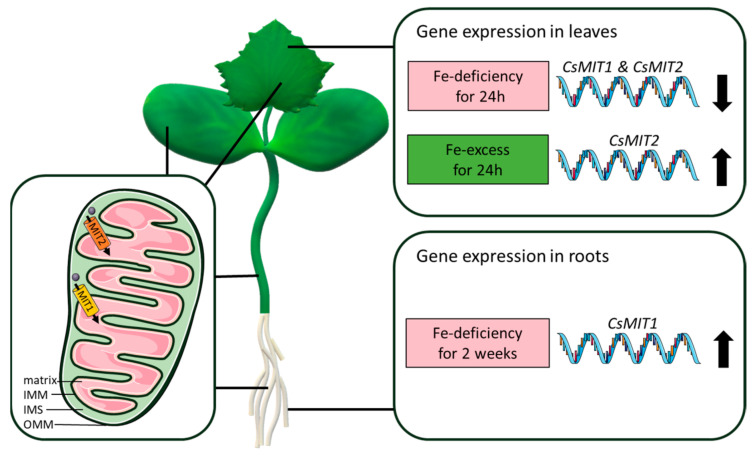
Possible role of mitoferrins in cucumber cells and their gene expression under different iron availability.

Reassuming, we have shown that two mitoferrins, CsMIT1 and CsMIT2, are present in cucumber cells. The obtained data suggest that both proteins, present in all the analyzed vegetative organs of cucumber, function as iron importers and deliver Fe from the cytoplasm to the mitochondria (Figure 6, left). Thus, like the previously characterized mitoferrins, they may play an important role in cellular and mitochondrial iron homeostasis, as mitochondria are a site of several crucial Fe-dependent metabolic pathways. Cucumber, like *Arabidopsis*, has two genes encoding mitochondrial mitoferrins, whereas potato, also a dicot plant, possesses only one. Studies using yeast mutants have shown that the cucumber proteins act redundantly. However, under variable iron availability, CsMIT1 and CsMIT2 are regulated differently at the gene expression level in roots and shoots. CsMIT1 appears to play an important role in roots under Fe-deficient conditions, while CsMIT2 may be relevant in leaves under Fe-excess conditions (Figure 6, right). Considering the differences in the expression profiles of mitoferrins studied thus far in plants exposed to varying iron availability, as well as the elemental profiles of *Arabidopsis* and rice mutant lines, it is important to extend our knowledge on iron homeostasis to many popular crop plants, including cucumber. This gained knowledge may, in turn, be later applied in widely understood agriculture.

Parts of the figure were drawn using pictures from Servier Medical Art Database. Servier Medical Art by Servier is licensed under a Creative Commons Attribution 3.0 Unported License (https://creativecommons.org/licenses/by/3.0/ (accessed on 27 February 2023)).

## Data Availability

The data presented are available in this manuscript and Supplementary Materials.

## Supplementary Materials

The following supporting information can be downloaded at: https://www.mdpi.com/article/10.3390/ijms24055050/s1.
