# Supplementary material for: Identification and Functional Analysis of Two Mitoferrins, CsMIT1 and CsMIT2, Participating in Iron Homeostasis in Cucumber
Source: Int J Mol Sci. 2023 Mar 6;24(5):5050. doi: 10.3390/ijms24055050 (PMC10003640; doi:10.3390/ijms24055050)
Supplement: Supplementary file 1 [file ijms-24-05050-s001.zip › ijms-2225037-supplementary.pdf]

Identification and functional analysis of two mitoferrins, CsMIT1 and CsMIT2, participating in iron homeostasis in cucumber

Karolina Małas [1] and Katarzyna Kabała [1,*]

Department of Plant Molecular Physiology, Faculty of Biological Sciences, University of Wrocław, Kanonia 6/8, 50-328 Wrocław, Poland

* Correspondence: katarzyna.kabala@uwr.edu.pl

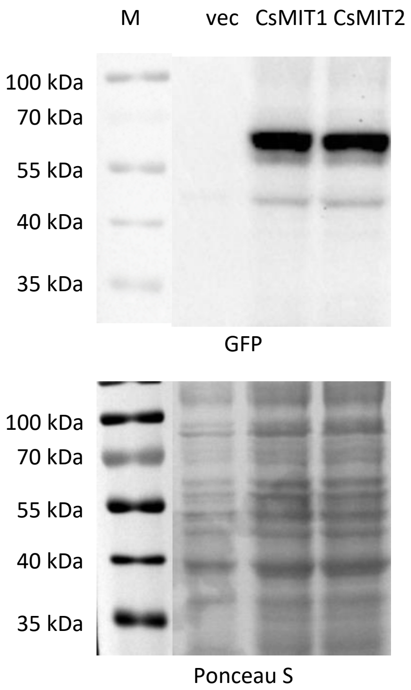

Figure S1- Immunoblotting of the mitochondria isolated from *Δmrs3Δmrs4* strain transformed with either pUG23-GFP (vec), pUG23-CsMIT1-GFP (CsMIT1) or pUG23-CsMIT2-GFP (CsMIT2) vector, using the antibodies against GFP.

Table S1. The sequences of primers used in this work. The restriction sites are underlined.

Primers	Sequences (5'-3')
Primers used for amplification of full coding sequence of CsMIT1 and CsMIT2	
**for ligation of CsMIT1 into pUG23 vector**	forward: AAATCTAGAATGGCTACCGAGGCGA
	reverse: TTTGAATTCGGTACCGTTGTGGCTGC
**for ligation of CsMIT2 into pUG23 or pUG35 vector**	forward: AAAGAATTCATGGCCACAAGCGTATC
	reverse: TTTGTCGACATTGTTGTGATTGTGGAGATGT
**for ligation of CsMIT1 into pA7-GFP vector**	forward: AAAACTAGTATGGCTACCGAGGCGA
	reverse: TTTACTAGTAGGTACCGTTGTGGCTGC
**for ligation of CsMIT2 into pA7-GFP vector**	forward: AAAGTCGACATGGCCACAAGCGTATC
	reverse: TTTACTAGTATTGTTGTGATTGTGGAGATGT
Primers used for real-time PCR	
**CsMIT1**	forward: CAATTAAATCTGTTGGAGTTCGAC
	reverse: ACTTCTTACAATTCTCGTAAACTG
**CsMIT2**	forward: CTTAGGTGGTGGATCGTCA
	reverse: CCTCTTTCGCAAACTCGTA
**CsCACS [51]**	forward: TGGGAAGATTCTTATGAAGTGC
	reverse: CTCGTCAAATTTACACATTGGT

Table S2. Strains and plasmids used in this work.

Strain	Genotype	Source or reference
**DY150**	*MATa ade2-1 can1-100 his3-11,15 leu2-3,112 trp1-1 ura3-52*	[54]
**DY150 *mrs3mrs4***	*mrs3::KanMX mrs4::KanMX*	[55]
**BY4742**	*MATα his3Δ1 leu2Δ0 lys2Δ0 ura3Δ0*	[56]
**BY4742 *zrc1***	*zrc1::KanMX4*	Euroscarf, Germany
**BY4742 *cot1***	*cot1::kanMX4*	Euroscarf, Germany
**BY4742 *ycf1***	*ycf1*:: *kanMX4*	Euroscarf, Germany
**BY4742 *ace1***	*ace1::KanMX4*	Euroscarf, Germany
**Plasmid**	**Description**	**Source or reference**
**pUG23**	*Escherichia coli*-yeast shuttle vector with the multiple cloning site (MCS) between the constitutive MET25 promoter and CYC1 termination sequence with C-terminal GFP reporter gene. HIS selectable marker and Amp^r	[52]
**pUG35**	*Escherichia coli*-yeast shuttle vector with the multiple cloning site (MCS) between the constitutive MET25 promoter and CYC1 termination sequence with C-terminal GFP reporter gene. URA selectable marker and Amp^r	[52]
**pA7-GFP**	pUC18-based vector for the transient expression of C-terminal GFP-fusion proteins in plant cells under the CaMV 35S promoter. Amp^r	[53]
**pRS426**	*Escherichia coli*-yeast shuttle vector carrying the *gentisate 1,2-dioxygenase* (*GDO*) gene from *Pseudaminobacter salicylatoxidans* tagged with FLAG epitope. URA selectable marker and Amp^r	kindly provided by Prof. Jerry Kaplan from University of Utah (USA)
